# A Larger Root System Is Coupled With Contrasting Expression Patterns of Phosphate and Nitrate Transporters in Foxtail Millet [*Setaria italica* (L.) Beauv.] Under Phosphate Limitation

**DOI:** 10.3389/fpls.2018.01367

**Published:** 2018-09-13

**Authors:** Zeeshan Ahmad, Faisal Nadeem, Ruifeng Wang, Xianmin Diao, Yuanhuai Han, Xingchun Wang, Xuexian Li

**Affiliations:** ^1^MOE Key Laboratory of Plant-Soil Interactions, Department of Plant Nutrition, China Agricultural University, Beijing, China; ^2^Institute of Crop Sciences, Chinese Academy of Agricultural Sciences, Beijing, China; ^3^Department of Crop Sciences, Shanxi Agricultural University, Taigu, China; ^4^College of Life Sciences, Shanxi Agricultural University, Taigu, China

**Keywords:** foxtail millet, phosphate limitation, root system, hormone accumulation, phosphate transporters, nitrate transporters

## Abstract

Foxtail millet [*Setaria italica* (L.) Beauv.], a widely cultivated food and fodder crop, develops a smaller root system while enlarges the root diameter facilitating nutrient transport under nitrogen limitation. How foxtail millet responds to phosphate limitation (LP) remains unaddressed. LP seedlings of the sequenced variety Yugu1 had significantly lower P concentrations in both shoots and roots and displayed higher levels of anthocyanin accumulation in leaves, indicating that the seedlings suffered from P limitation under hydroponic culture. One obvious and adaptive phenotype of LP plants was the larger root system mostly as the result of stimulation of lateral root proliferation in terms of the number, density, and length. Preferential biomass accumulation in the root under LP ensured carbon provision for root expansion and resulted in significant increases in the total and specific root length, which substantially extended the absorptive surface of P in the growth medium. Elevation of auxin and gibberellin concentrations might serve as an internal boost underpinning root architectural re-patterning under LP. Not just morphological adaptation, up-regulation of expression of *SiPHT1;1* and *SiPHT1;4* in roots and that of *SiPHT1;2* in roots and shoots preconditioned adaptive enhancement of P uptake and translocation under LP. Interestingly, internal nitrogen surpluses occurred as indicated by dramatic increases in free amino acids in LP shoots and roots and higher concentrations of nitrogen in roots. Such nitrogen surplus ‘signals’ tended to switch down expression of nitrate transporters *SiNRT2.1* and *SiNAR2.1* in the root and that of *SiNRT1.11* and *SiNRT1.12* in the shoot to reduce nitrate mobilization toward or within the shoot. Together, our work provided new insights into adaption of a critical cereal crop to LP and its innate connection with nitrogen nutrition.

## Introduction

For a typical plant, the phosphorus (P) concentration is about 1 μM in the soil, 400 μM in the xylem, and 10,000 μM in the cytoplasm (Fang et al., 2009). P is utilized by plants for numerous functions including photosynthesis, respiration, energy generation, nucleic acid synthesis, glycolysis, redox reactions, membrane synthesis and stability, and nitrogen fixation (Abel et al., 2002; Vance et al., 2003). Despite its importance for plants, phosphate bio-availability in soil solution is very low due to its tendency to bind strongly to soil surfaces or form pH-dependent insoluble complexes with cations (Shen et al., 2011). P deficiency is one of the greatest limitations in agricultural production (Schachtman et al., 1998; Lynch and Brown, 2008). It has been estimated that 5.7 billion hectares of agricultural land is deficient in phosphorus worldwide (Cordell et al., 2009). Continuous provision of P fertilizers is required to sustain high productivity levels (Schachtman et al., 1998). Phosphate rock, the primary source of inorganic phosphorus fertilizers, is minable in only a few areas in the world and likely becomes more costly when approaching toward peak phosphorus demand around 2030 (Cordell et al., 2009).

Plants have evolved a complex array of strictly regulated mechanisms to maintain P homeostasis under P deficient conditions. First, the modification of root architecture is a powerful tool of plants for high P acquisition (Lynch, 1995). Typical morphological responses to P limitation (LP) include a highly branched root system with more and longer root hairs and/or associations of mycorrhizal fungi, which enlarges the total surface area for soil exploration and P acquisition (Raghothama, 1999; Vance et al., 2003; Lambers et al., 2006). Upon LP, white lupin develops densely branched cluster roots (Gardner et al., 1982); *Phosphorus uptake 1* (*Pup1*), a major QTL conferring rice tolerance to P deficiency in the soil, promotes crown root proliferation in rice (Wissuwa et al., 1998, 2002; Gamuyao et al., 2012). In rice and *Brassica oleracea*, P uptake is correlated with the lateral root number, lateral root length, and root growth rate under LP (Li et al., 2007; Hammond et al., 2009); or plants increase number and/or length of lateral roots and root hairs to enhance P capture (Raghothama, 1999; Lynch, 2011). Second, hormone synthesis, transport, and signaling may be affected by nutrient limitations, which in turn cause morphological, physiological, and molecular alterations in the root (Torrey, 1976; Malamy, 2005; Osmont et al., 2007; Kiba et al., 2010). Auxin plays a fundamental role in regulating root development (Sabatini et al., 1999), and blockage of auxin synthesis or signaling causes severe developmental defects in roots (Went and Thimann, 1937). P limitation alters auxin transport, distribution, or sensitivity to promote lateral root development in *Arabidopsis* (López-Bucio et al., 2005; Nacry et al., 2005). Gibberellin regulates organ differentiation (Yamaguchi, 2008). Third, phosphate transporters, *i.e.*, PHT1, PHT2, PHT3, and PHT4 families, play vital roles in P acquisition and translocation (Rausch and Bucher, 2002). Among these four transporter families, the PHT1 family is most widely studied and most PHT1 members are specifically or preferentially expressed in root epidermis cells primarily under the regulation of the cellular P concentration (Mudge et al., 2002; Rae et al., 2003).

Foxtail millet [*Setaria italica* (L.) Beauv.] was domesticated from *Setaria viridis* in northern China between 8700 and 5900 years ago (Barton et al., 2009). At present, foxtail millet is cultivated in 26 countries and ranks second in world’s millet production (Li and Wu, 1996; Yang et al., 2012). Release of the complete genome facilitates functional and evolutionary investigation of foxtail millet as a model crop species (Li and Brutnell, 2011; Bennetzen et al., 2012; Zhang et al., 2012; Daverdin et al., 2015). Foxtail millet develops a smaller root system under nitrogen limitation, enlarges the root diameter, and up-regulates expression of nitrogen transporters for enhanced nitrogen uptake and translocation (Nadeem et al., 2018). It remained an intriguing question how foxtail millet responds to LP? Our hydroponic experiments provided insights into adaptations of root architecture, hormone and metabolite accumulation, and expression of transporters to LP and their connection with nitrogen nutrition in foxtail millet seedlings.

## Materials and Methods

Seeds of foxtail millet (the sequenced variety Yugu1) (Cheng and Dong, 2010) were washed three times with deionized water, sterilized for half an hour with 10% H_2_O_2_, imbibed in saturated CaSO_4_ solution for 5 h, and germinated on moist filter paper. Seedlings with 2-cm roots were wrapped in moist filter paper and placed vertically in the growth holder saturated by deionized water and covered with black plastic until leaf emergence. Uniform seedlings having fully expanded leaves were grown in the greenhouse of China Agricultural University, Beijing, P. R. China (temperature 26/20°C; photoperiod 14/10 h day/night; relative humidity 45–55%). The whole nutrient solution as control (CK) consisted of 2 mM NH_4_NO_3_, 0.25 mM KH_2_PO_4_, 0.75 mM K_2_SO_4_, 0.1 mM KCl, 2 mM CaCl_2_, 0.65 mM MgSO_4_, 0.2 mM Fe-EDTA, 1 × 10^-3^ mM MnSO_4_, 1 × 10^-3^ mM ZnSO_4_, 1 × 10^-4^ mM CuSO_4_, 5 × 10^-6^ mM (NH_4_)_6_Mo_7_O_24_, 1 × 10^-3^ mM H_3_BO_3_. 25% nutrient solution was applied for the first three days, 50% nutrient solution for next four days and 100% nutrient solution for 1 week. Seedlings were subjected to LP during the third week of millet growth. To make the LP medium, 0.25 mM KH_2_PO_4_ was reduced to 1% CK, while 0.1 mM KCl was used to replenish potassium concentration in the LP nutrient solution while other nutrients remained unchanged. The pH was maintained at 6.0. Every 3.4-L continuously aerated pot containing four seedlings represented one biological replicate. Each treatment had six biological replicates and the nutrient solution was changed every 2 days.

SPAD values were measured before harvest with a Chlorophyll Meter (SPAD-502, Konica Minolta Sensing Inc., Japan). The 4th leaf (the youngest fully expanded leaf) of all four plants in each pot was analyzed three times with the SPAD meter. Average of three SPAD values from one leaf represented one read and average of four reads from one pot was taken as one biological replicate. Root and shoot samples were harvested on the 21st day after transfer to the nutrient solution. Samples were carefully washed three times with deionized water, gently wiped with blot paper, immediately frozen in liquid N_2_, and stored at -80°C for physiological measurements. Samples were harvested and washed three times, oven-dried at 105°C for 30 min, then dried at 70°C until constant weight for dry weight (DW) and other related analysis.

### Analysis of P and N Concentrations and the C/N Ratio

Oven-dried shoot and root tissues were ground into fine powder and digested with H_2_SO_4_-H_2_O_2_ followed by P analysis using spectrophotometer at 440 nm by the modified vanadomolybdate method (Johnson and Ulrich, 1959), and total N analysis using a modified Kjeldahl digestion method (Baker and Thompson, 1992). The C/N ratio was analyzed by loading ∼50 mg fine shoot or root powder into the Elementar vario Macro CN analyzer (Elementar Technologies, Hanau, Germany).

### Biochemical Analysis

Fresh leaves were weighed and ground in liquid nitrogen, dissolved in the anthocyanin isolation solution (methanol: concentrated hydrochloric acid, 99:1v/v). Spectrophotometer was used to determine OD530 and OD657 values (Rabino and Mancinelli, 1986). The concentration of soluble sugars was determined using a commercially available kit (Boehringer Mannheim, Germany). The concentration of total free amino acids was measured according to the Rosen ninhydrin colorimetric method by using leucine as a standard (Rosen, 1957). A standard kit (Coomassie Protein assay reagent; Bio-Rad, Hercules, CA, United States) was used as a reference to extract and analyze soluble proteins, with bovine serum albumin as the reference.

### Root Architecture

The whole root was well spread and scanned with a scanner (Epson 1680, Indonesia). The scanned images were analyzed using the WinRHIZO software (version 5.0) (Regent Instruments Inc., Quebec City, QC, Canada) to get the total root length following the previously described method (Peng et al., 2010). Each type of root was counted manually. The crown root length was measured with a ruler. The lateral root density was defined as the number of lateral roots per unit of the crown root length.

### Hormone Extraction and Quantification by Enzyme Linked Immunosorbent Assay (ELISA)

Fresh shoot and root samples (0.5 g) were homogenized individually in 2 mL of 80% methanol (containing 40 mg butylated hydroxytoluene as antioxidant). This mixture was incubated at 4°C for 48 h, and then centrifuged at 1900 ×*g* at 4°C for 15 min. The supernatant was passed through C18 Sep-Pak cartridges (Waters Corp), and the hormone fraction was eluted with 10 mL of 100% (v/v) methanol and then 10 mL ether. The elute was N_2_-dried at 20°C. The N_2_-dried extracts were dissolved in 2.0 mL phosphate-buffered saline (PBS) containing 0.1% (v/v) Tween-20 and 0.1% (w/v) gelatin (pH 7.5) to analyze the concentration of free indole-3-acetic acid (IAA), zeatin riboside (ZR), gibberellin (GA3), and abscisic acid (ABA) by ELISA following a well-established protocol (Weiler et al., 1981).

### RNA Isolation and Quantitative Real-Time PCR

Total RNA was extracted from root and shoot samples by using TRIzol reagent according to the manufacturer’s instructions (Invitrogen). RNA samples (4–5 g) were digested by DNase I (Takara Biomedicals, Kyoto, Japan) to remove any potential DNA contamination, then reverse transcribed into cDNA using M-MLV reverse transcriptase (Invitrogen). Gene expression levels were determined by quantitative real-time PCR in a Bio-Rad iCycler iQ5 system (Bio-Rad, Hercules, CA, United States) using the SYBR Premix Ex Taq^TM^ (Takara) and gene specific primers (**Supplementary Table S1**). The program was 10 min pre-incubation at 95°C, followed by 40 cycles of denaturation at 95°C for 15 s, annealing at 60°C for 30 s, and extension at 72°C for 30 s. The relative gene expression levels were calculated following the standard comparative method (Livak and Schmittgen, 2001). Each treatment had three biological replicates, with three technical replicates for every biological replicate.

### Statistical Analysis

Data were analyzed using the one-way ANOVA in Statistix 8.1 (Analytical Software, 2005). Means of different treatments were compared using the least significant difference at a 0.05 level of probability.

## Results

### Physiological Responses of Foxtail Millet to LP

**FIGURE 1:**
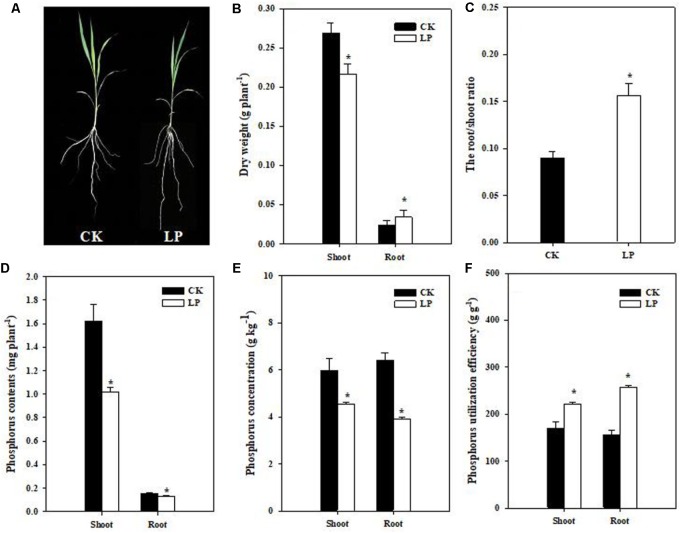
Shoot and root responses of foxtail millet [*Setaria italica* (L.) Beauv.] to LP. Plant growth with intact root and shoot **(A)**, shoot and root dry weight (g plant^-1^) **(B)**, the root/shoot ratio **(C)**, P accumulation **(D)**, phosphorus concentration **(E)**, P utilization efficiency **(F)**. Each bar represented SE of six biological replicates. ^∗^indicated significant difference between treatments (*P* < 0.05).

Seedlings of foxtail millet were exposed to P limitation (LP) condition for 1 week. Compared to control (CK) plants, LP plants had a relatively larger root system (**Figure 1A**). P deficiency caused a 37.5% increase in the root dry weight while significantly reduced biomass accumulation in the shoot as compared to the control. As a result, the root/shoot ratio increased by 77.78% (**Figures 1B,C**). LP reduced P accumulation in the shoot and root, with a larger decrease in the P concentration in the root than in the shoot (**Figures 1D,E**). We defined P utilization efficiency (PUtE) as cumulative biomass per unit of P (g DW g^-1^) and found 30.46% higher PUtE in the shoot and 63.27% higher in the root under LP (**Figure 1F**).

**FIGURE 2:**
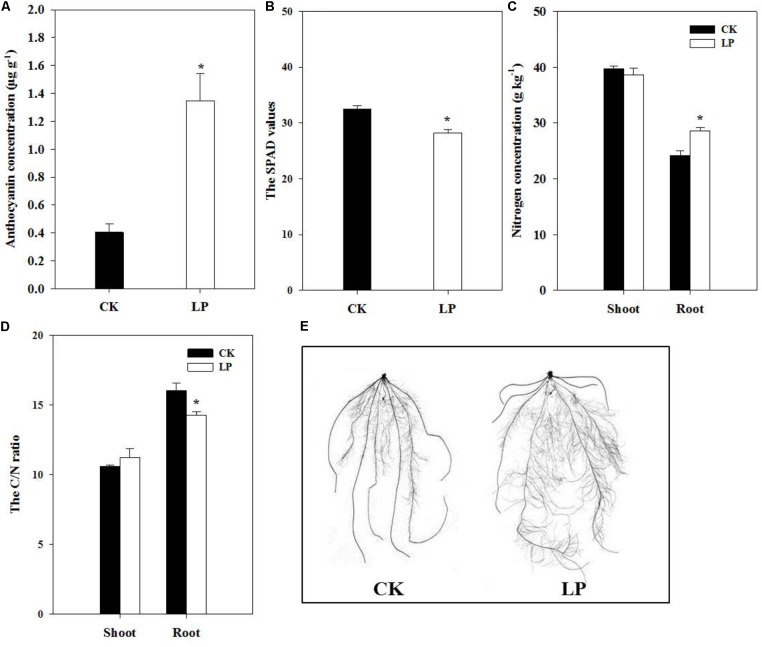
Biochemical and morphological responses of foxtail millet to LP. The anthocyanin accumulation in leaves **(A)**, the SPAD value **(B)**, nitrogen concentration in the shoot and root **(C)**, the C/N ratio **(D)**, scanned root systems **(E)**. Each bar represented SE of six biological replicates. ^∗^indicated significant difference between treatments (*P* < 0.05).

Visible accumulation of anthocyanin pigmentation is a characteristic response of plants to P starvation. We observed enhanced accumulation of anthocyanin (1.35 μg g^-1^) in leaves of LP-seedlings (**Figure 2A**). Interestingly, P deficiency caused significant decreases in the SPAD value of the fourth leaf at harvest (**Figure 2B**) in contrast to a significant increase in the nitrogen concentration in the root (**Figure 2C**) and a consequent decrease in the C/N ratio of the LP root (**Figure 2D**). In spite of lower SPAD values under LP, there was no significant difference in the concentration of soluble sugars in plants under LP and control conditions (**Table 1**). On the other hand, LP resulted in increases in the concentration of free amino acids by 83.87% in the shoot and 104.17% in the root (**Table 1**) and decreases in accumulation of soluble protein in the root (21%) and shoot (24.76%) (**Table 1**).

**Table 1:** Percentage changes in biochemical parameters in the shoot and root.

Biochemical parameters	Plant tissue	Treatment	Concentration (mg g^-1^)	Percentage change (%)
Total soluble	Shoot	CK	3.94 ± 0.11	ns
sugars		LP	4.04 ± 0.28	
	Root	CK	3.21 ± 0.31	ns
		LP	3.14 ± 0.38	
Free amino	Shoot	CK	0.31 ± 0.01	83.87
acids		LP	0.57 ± 0.01*	
	Root	CK	0.24 ± 0.01	104.17
		LP	0.49 ± 0.01*	
Total soluble	Shoot	CK	5.29 ± 0.24	-24.76
proteins		LP	3.98 ± 0.35*	
	Root	CK	3.19 ± 0.13	-21
		LP	2.52 ± 0.02*	


*Asterisks indicated significant differences (*P* < 0.05) between “CK” and “LP.” Percentage change = [(value under LP-value under CK)/value under CK]^∗^100%.*

### Root Architectural Alterations Under LP

The plasticity of root architecture is a developmental advantage of plants in response to environmental stresses. Plants may undergo dramatic root morphological alterations to enhance P foraging capacity. To quantify changes in root architecture due to P deficiency, we scanned the root system of foxtail millet at harvest (**Figure 2E**). LP caused no difference in the number of crown roots but increased the number of lateral roots by 58.62%, crown root length by 23.9%, lateral root length by 73.25%, lateral root density (the ratio of the lateral root number to the crown root length) by 27.2%, total root length by 67.35%, and specific root length (the ratio of the total root length to root biomass) by 21.39% compared to the control (**Table 2**), indicating an overall stimulatory effect of LP on lateral root development and longitudinal growth of the entire root system.

**Table 2:** Percentage changes in root measurements of foxtail millet.

Root parameter	Treatment	Quantification	Percentage change (%)
Crown root	CK	8.16 ± 0.65	
number	LP	8.33 ± 0.42	ns
Crown root	CK	132.67 ± 7.08	
length (cm)	LP	164.33 ± 7.89*	23.9
Lateral root	CK	683.76 ± 17.89	
number	LP	1084.5 ± 51.11*	58.62
Lateral root	CK	978.59 ± 39.60	
length (cm)	LP	1695.46 ± 66.94*	73.25
Lateral root	CK	5.22 ± 0.28	
density (No. cm^-1^)	LP	6.64 ± 0.34*	27.2
Total root	CK	1111.25 ± 33.61	
length (cm)	LP	1859.78 ± 68.63*	67.35
Specific root	CK	46652.62 ± 1069.76	
length (cm g^-1^)	LP	56629.83 ± 2663.65*	21.39


*Asterisks indicated significant differences (*P* < 0.05) between “CK” and “LP.” Percentage change = [(value under LP-value under CK)/value under CK]^∗^100%.*

### Changes in Hormone Accumulation Under LP

**FIGURE 3:**
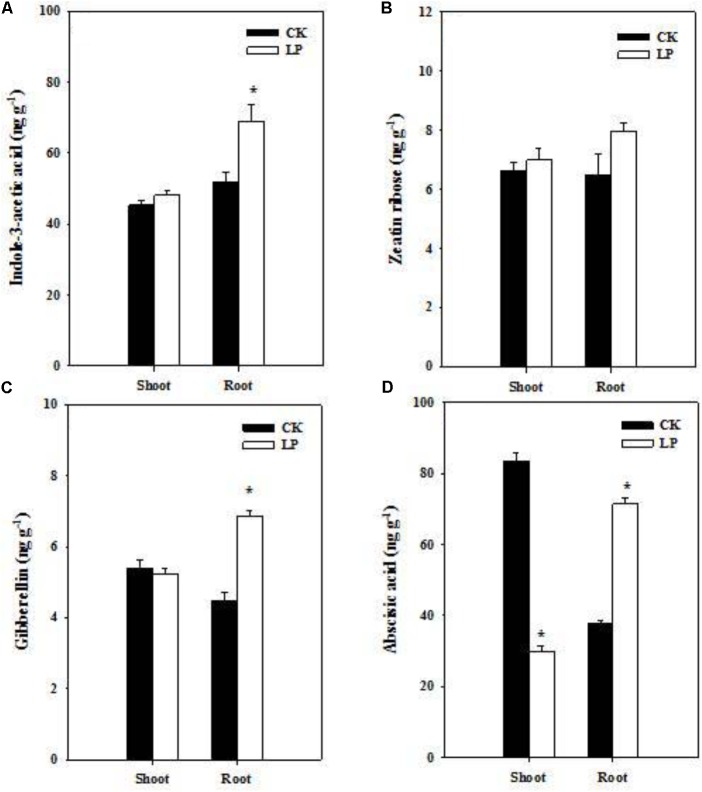
Hormonal responses of foxtail millet to LP. Indole-3-acetic acid **(A)**, zeatin ribose **(B)**, gibberellin **(C)**, abscisic acid **(D)** in the shoot and root. Each bar represented SE of six biological replicates. ^∗^indicated significant differences between treatments (*P* < 0.05).

Hormones are crucial regulators of plant growth and development under frequently changing environmental conditions (Marsch-Martinez and de Folter, 2016). Our results showed distinct effects of LP on hormone accumulation in the root and shoot (**Figure 3**). LP led to increases in the concentration of auxin and gibberellin (GA3) in the root (**Figures 3A–C**). P withdrawal from the nutrient solution did not change IAA and GA3 concentrations in the shoot as well as the zeatin riboside concentration in the shoot and root (**Figures 3A–C**). The ABA concentration increased by 88.3% in the root while decreased by 64% in the shoot under P deficient conditions (**Figure 3D**).

### Expression Analysis of Phosphate and Nitrogen Transporters by Quantitative Real Time RT-PCR

**FIGURE 4:**
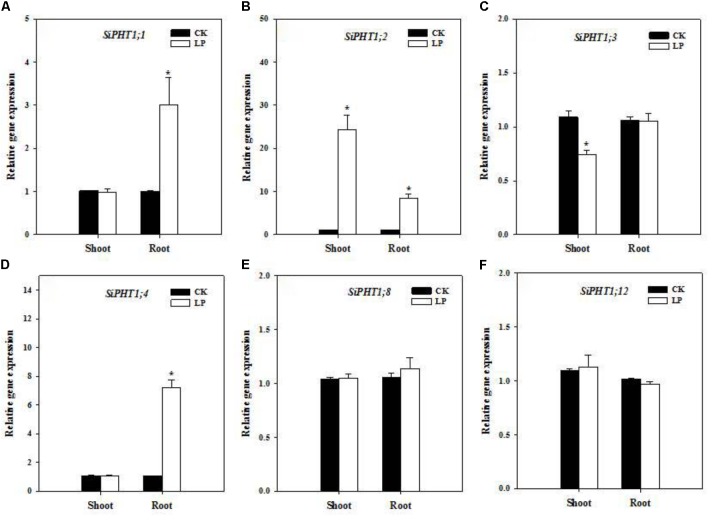
Transcript abundance of phosphate transporters. Relative expression of *SiPHT1;1* **(A)**, *SiPHT1;2* **(B)**, *SiPHT1;3* **(C)**, *SiPHT1;4* **(D)**, *SiPHT1;8* **(E)**, *SiPHT1;12* **(F)** in the shoot and root under CK and LP. Each bar represented SE of three biological replicates. ^∗^indicated significant differences between treatments (*P* < 0.05).

Enhanced nutrient uptake or translocation is another crucial mechanism for plants to encounter nutrient limitation in addition to root morphological adaption. Phosphate transporters mediate P uptake from the growth medium and translocation within the plant and expression of a series of phosphate transporters is up-regulated under P limitation (Leggewie et al., 1997). We identified a subset of phosphate transporters in foxtail millet (gene IDs were listed in **Supplementary Table S1**) and analyzed their expression levels in the root and shoot by quantitative real time PCR. *SiPHT1;1* expression was up-regulated in roots (**Figure 4A**). *SiPHT1;2* showed a dramatic upregulation of expression in shoots (>20 fold) and roots (>8 fold) (**Figure 4B**) while *SiPHT1;3* expression was down-regulated in shoots (**Figure 4C**), whereas, *SiPHT1;4* expression was also up-regulated in roots (∼7 fold) (**Figure 4D**). No change was observed in expression patterns of *SiPHT1;8* and *SiPHT1;12* under LP (**Figures 4E,F**).

**FIGURE 5:**
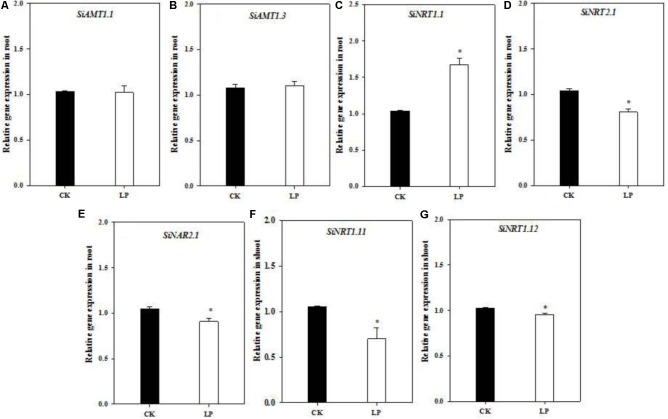
Quantitative real-time PCR analysis of nitrogen transporters. Relative expression of *SiAMT1.1* **(A)**, *SiAMT1.3* **(B)**, *SiNRT1.1* **(C)**, *SiNRT2.1* **(D)**, *SiNAR2.1* **(E)**, in the root and *SiNRT1.11* **(F)**, *SiNRT1.12* **(G)** in the shoot under CK and LP. Each bar represented SE of three biological replicates. ^∗^indicated significant differences between treatments (*P* < 0.05).

One interesting question is whether over-accumulation of amino acids and a large root system under LP affect expression of nitrogen transporters involved in nitrogen uptake and allocation? To this respect, we analyzed transcript abundance of nitrogen transporters involved in nitrogen uptake, transport or remobilization (gene IDs were listed in **Supplementary Table S1**; Nadeem et al., 2018) under control and LP conditions. The expression level of *SiAMT1.1* and *SiAMT1.3* did not change in roots (**Figures 5A,B**). SiNRT1.1 expression was dramatically up-regulated in the root grown under LP compared to the control (**Figure 5C**). Transcript abundance of *SiNRT2.1* and *SiNAR2.1* in the roots and that of *SiNRT1.11* and *SiNRT1.12* in shoots was significantly reduced under LP (**Figures 5D–G**).

## Discussion

Phosphate is easily fixed in the soil across different eco-zones worldwide, which makes P a low bio-available macronutrient (Hinsinger, 2001; Shen et al., 2011). To promote P acquisition, plants reshape root architecture to favor P capture, modulate transporter systems to facilitate P influx and remobilization, release carboxylates to activate fixed phosphate, and/or allow colonization of mycorrhizal fungi to enhance P forage (Jakobsen et al., 1992; Bates and Lynch, 1996; Jeschke et al., 1997; Hinsinger, 2001; Williamson et al., 2001; Vance et al., 2003; Ticconi and Abel, 2004; Bucher, 2007). Foxtail millet is a large-root crop and responds to nitrogen limitation by reducing its total root length and increasing the root diameter presumably for higher transport efficiencies (Nadeem et al., 2018). How foxtail millet physiologically responds to LP becomes an interesting question to be addressed.

### P Limitation Stimulated Root Growth in Foxtail Millet

The LP treatment significantly reduced P accumulation in the shoot and root, and the shoot displayed discernible LP symptoms (higher levels of anthocyanins; **Figure 2A**), indicating that seedlings suffered from P limitation. One obvious and adaptive phenotype of LP plants was the larger root system (**Figure 2E** and **Table 2**). Root architecture generally refers to the shape and structure of a root system programmed by intrinsic developmental regulators and external environmental cues in a well coordinative manner (Hodge et al., 2009; Wei and Li, 2016). P uptake by plants heavily depends on the total length and surface area of the root in a given soil volume (Richardson et al., 2009; Balemi and Negisho, 2012; Lambers et al., 2015). In contrast to overall inhibitory effects of N limitation on root growth (Nadeem et al., 2018), LP promoted lateral root proliferation in terms of number, density, and length in foxtail millet (**Table 2**) in contr. Such multilevel stimulation of lateral root growth generated a highly branched root structure, and more importantly enlarged the absorptive surface area (Bates and Lynch, 1996; Williamson et al., 2001; López-Bucio et al., 2002; Pérez -Torres et al., 2008; Péret et al., 2011). The length of crown roots increased under LP although their number remained a similar level to the control (**Table 2**). As a result, the total root length increased by 67.35% (**Table 2**), which substantially improved capabilities of P capture in the growth medium at the morphological level. Supporting root growth, preferential biomass accumulation in the root occurred under LP at the expense of shoot growth (**Figures 1B,C**), which ensured carbon provision for further root expansion and resulted in an ultimate significant increase in the specific root length (**Table 2**). On the other hand, root patterning is orchestrated by internal regulators in the LP-triggered signaling cascade. Auxin and gibberellin signaling plays critical roles in root genesis and patterning (Went and Thimann, 1937; Sabatini et al., 1999; Yamaguchi, 2008). P availability-dependent hormone functioning in crop growth and development has been frequently documented (Rubio et al., 2009); however, many observations are not so conclusive at physiological levels (Borch et al., 1999; Kumar et al., 2014). Notably, concentrations of auxin and gibberellin increased in roots rather than in shoots (**Figures 3A,C**). Over-accumulation of these two hormones in roots boosted lateral root proliferation and elongation of the whole root system and gave rise to many other profound biological consequences to be further investigated. ABA could function as a promoter in lateral root development under nutrient limitation (De Smet et al., 2003). Higher levels of ABA under LP might have a synergistically stimulatory role in root development (**Figure 3D**) in addition to its primary role in the stress response.

### P Limitation Caused Up-Regulation of Expression of Phosphate Transporters and P Utilization Efficiency

Root morphological adaptation was a primary strategy of foxtail millet to cope with P limitation (**Figure 2E** and **Table 2**), by which the root grew substantially larger in terms of the absorptive surface area. Ultimate physiological outcomes of root enlargement heavily relied on the functionality of phosphate transporters in the root and shoot. Expression of many PHT1 members is stimulated by P limitation (Mudge et al., 2002; Rae et al., 2003). *OsPHT1*;*2* expression is strongly induced in roots (especially the stele and lateral roots) by LP presumably to enhance P uptake through the root and translocation to the shoot although *OsPHT1*;*1* expression is independent of P levels (Shrawat and Lörz, 2006; Ye et al., 2015). *OsPHT1*;*4*, primarily expressed in the root and embryo, shows up-regulation in the shoot under P deficient conditions (Ye et al., 2015; Zhang et al., 2015). Phosphate transporters are broadly conserved across cereal crops (Rakshit and Ganapathy, 2014). According to phylogenetic analysis, *SiPHT1;2* is homologous to high affinity transporter *OsPHT1*;*8* that is involved in source-to-sink P mobilization (Jia et al., 2011; Ceasar et al., 2014), with *SiPHT1;3* homologous to *OsPHT1*;*4* and *OsPHT1*;*5* and *SiPHT1*;*4* homologous to *OsPHT1*;*1* and *OsPHT1*;*2* (Ceasar et al., 2014). Our results showed significant up-regulation of expression of *SiPHT1;1* and *SiPHT1;4* in root tissues (**Figures 4A,D**), presumably preconditioning enhanced P uptake from the root.

Interestingly, *SiPHT1;3* expression was down-regulated in the shoot under LP (**Figure 4C**). Down-regulation of *SiPHT1;3* expression probably favored P retention in the shoot or allocation toward nascent leaves. Although LP-plants had larger root systems and higher levels of expression of P transporters, phosphate in the nutrient solution was so limited that stressed seedlings inevitably took advantage of the internal P reserve, which resulted in decreases in total P accumulation and P concentration, and higher PUtE (**Figures 1D–F**), in agreement with previous studies with different crops (Barker and Pilbeam, 2007; Rose et al., 2011).

### P Limitation Altered Accumulation of Nitrogen and Nitrogen Metabolites and Expression of Nitrogen Transporters

Beyond root morphological alterations and differential expression of phosphate transporters, LP plants had higher nitrogen concentrations and lower C/N ratios in the root (**Figures 2C,D**), indicating that P limitation caused elevation of nitrogen accumulation in the root. Further, the concentration of free amino acids approximately doubled in the shoot and root of LP-seedlings probably due to enhanced protein degradation, as indicated by decreases in concentrations of soluble proteins in the shoot and root (**Table 1**). Our results were consistent with previous reports on increases in accumulation of amino acids under P deficiency (Rufty et al., 1990, 1993; Huang et al., 2008). Indeed, protein degradation is stimulated while photosynthesis and protein synthesis are down-regulated at the transcription level in P deficient *Arabidopsis* (Wu et al., 2003; Misson et al., 2005). In this context, lower levels of chlorophylls (indicated by SPAD values) in LP-leaves (**Figure 2B**) contributed to reduction of overall photosynthesis, thus biomass accumulation in the shoot decreased (Brahim et al., 1996).

The crucial function of a root system is to absorb nutrients from the growth environment for photosynthesis, growth and development, and biomass accumulation, which requires a large quantity of nitrogen influx and subsequent metabolism and accumulation (Miller et al., 2007, 2009; Tsay et al., 2007; Forde and Walch-Liu, 2009; Gojon et al., 2009; Krouk et al., 2010). However, nitrate uptake in the root is regulated by shoot-derived HY5 and CLE peptides (Jonassen et al., 2008; Tabata et al., 2014; Chen et al., 2016). Likewise, nitrogen capture of LP-seedlings was also modulated by shoot-derived signals. Nitrogen surplus ‘signals’ putatively generated by higher concentrations of free amino acids in the shoot tended to down-regulate nitrogen uptake. Nitrate uptake mediated by a sophisticated sensing and transport system is a critical step in nitrogen acquisition under frequently changing external environments (Dechorgnat et al., 2011; Wang et al., 2012). Among nitrate transporters, NRT1.1 functions as a nitrate sensor and dual-affinity nitrate transporter (Liu et al., 1999; Krouk et al., 2006; Ho et al., 2009). NRT2.1 serves as a high-affinity nitrate transporter whereas NAR2.1 is an assessory protein closely related to NRT2.1 functioning (Okamoto et al., 2006; Orsel et al., 2006; Tsay et al., 2007; Miller et al., 2009). As expected, expression of both *SiNRT2.1* and *SiNAR2.1* was significantly down-regulated in the root of LP-plants (**Figures 5D,E**). Alternatively, P and nitrogen signals are integrated by nitrate inducible GARP-type transcriptional repressor 1 (NIGT1) in *Arabidopsis*, and PHR1 promotes expression of *NIGT1*-clade genes under LP which in turn down-regulate *NRT2.1* expression (Maeda et al., 2018). Further, the expression of *SiNRT1.11* and *SiNRT1.12* was also down-regulated in the shoot of LP-plants (**Figures 5F,G**), likely reducing xylem-to-phloem transfer and within-shoot remobilization of nitrate (Hsu and Tsay, 2013). As a result, more nitrogen retained in the LP-root (**Figure 2C**). On the other hand, a larger root under LP somehow favored up-regulation of expression of *SiNRT1.1* (probably as a low-affinity transporter in this context) in the root (**Figure 5C**).

## Conclusion

Under P deficiency, a larger root developed primarily via stimulation of lateral root proliferation in terms of the number, density, and length in foxtail millet [*Setaria italica* (L.) Beauv.]. Preferential carbon provision and elevation of auxin and GA3 accumulation in the LP-root might serve as an internal boost for root morphological enlargement. Up-regulation of expression of *SiPHT1;1*, *SiPHT1;2*, and *SiPHT1;4* favored the functionality of the LP-root for P uptake and translocation. Interestingly, LP enhanced accumulation of amino acids and caused down-regulation of expression of nitrate transporters *SiNRT2.1*, *SiNAR2.1*, *SiNRT1.11*, and *SiNRT1.12*, indicating complicated connections between P and nitrogen nutrition. Exploration of underlying molecular mechanisms of P and nitrogen interaction holds the promise for breeding dual-nutrient efficient crop varieties in the future.

## Author Contributions

XL, ZA, and XD designed the research. ZA, FN, and RW performed the research. XL, ZA, YH, and XW analyzed the data. ZA and XL wrote the paper. FN, XD, YH, and XW revised the manuscript. All authors approved the final manuscript.

## Conflict of Interest Statement

The authors declare that the research was conducted in the absence of any commercial or financial relationships that could be construed as a potential conflict of interest.
