# Supplementary material for: A Larger Root System Is Coupled With Contrasting Expression Patterns of Phosphate and Nitrate Transporters in Foxtail Millet [Setaria italica (L.) Beauv.] Under Phosphate Limitation
Source: Front Plant Sci. 2018 Sep 13;9:1367. doi: 10.3389/fpls.2018.01367 (PMC6146770; doi:10.3389/fpls.2018.01367)
Supplement: Supplementary file 1 [file Table_1.DOC]

| **Supplementary Table | G**ene ID and related information for qRT-PCR analysis. | | | | |
| --- | --- | --- | --- | --- |
| **Gene Symbol** | **Description** | **Gene ID** | **Primer sequence** | **Product size (bp)** |
| *SiPHT1;1* | Phosphate transporter 1;1 | Si035047m.g | F: CTCATCCTCTCCACGGTGTT  R: CCGCCAGTAGTAGGTCAGGA | 150 |
| *SiPHT1;2* | Phosphate transporter 1;2 | Si035059m.g | F: ACCAGGACAAGAGCAAGGTG  R: GGCACGAGGAACGTGAGTAT | 115 |
| *SiPHT1;3* | Phosphate transporter 1;3 | Si011707m.g | F: TGTCATCGGGTTCTTGTTCA  R: AATTGGTCGGAACAGTCTGC | 128 |
| *SiPHT1;4* | Phosphate transporter 1;4 | Si035143m.g | F: CAGAAGGAGATCCAGGACGA  R: CGATATCGAGCAGGAACCAC | 145 |
| *SiPHT1;8* | Phosphate transporter 1;8 | Si033125m.g | F: GGATACTCTTCACCTGCTTCCT  R: TACAGCGGTAGAATCTGGGAGT | 133 |
| *SiPHT1;12* | Phosphate transporter 1;12 | Si006153m.g | F: GTCACCTTCTACTGGAGGATGG  R: TGTAGTAGGGGATGTCCAGGAG | 289 |
| *SiAMT1.1* | Ammonium transporter 1.1 | Seita.1G237300 | F: GTCCTTCCTCACCATCCT  R: CGTTCCAGTGCCCCGTCT | 157 |
| *SiAMT1.3* | Ammonium transporter 1.3 | Seita.1G189700 | F: CCCAAACTCGAGAGGTGCAT  R: TGCACATTCCACATTCCTCCA | 194 |
| *SiNRT1.1* | Nitrate transporter 1.1 | Seita.9G327900 | F: TGACTTGGTTTTGGCGTGTA  R: ACGCTTCCATTCATTCCAAG | 200 |
| *SiNRT2.1* | Nitrate transporter 2.1 | Seita.1G115700 | F: CCTGCTGTTTGTGTTTGTGC  R: TGAACCCTTGTGCACCTACT | 187 |
| *SiNAR2.1* | Nitrate assimilation related 2.1 (NAR2.1/NRT3.1) | Seita.1G218500 | F: GACAAGGCGTGCCAGTTC  R: CGTAGTAGGTGCCCGAGG | 106 |
| *SiNRT1.11* | Phloem transporter 1.11 | Seita.3G406400 | F: AATCGCCAAGTGCTATGGTC  R: CATGACAGCAGAAGCAGAGC | 220 |
| *SiNRT1.12* | Phloem transporter 1.12 | Seita.3G243200 | F:TCAAGTAGCTTGGTGGTTGCT  R: ACTCCTGCATTTCTCGAACA | 170 |
| *EF-Iα | Elongation factor 1-alpha | Si022040m.g | F: CAACAAGATGGATGCCACCAC  R: GAGATTGGGACGAAGGCAATC | 126 |
